# Alterations in Spontaneous Neuronal Activity and Microvascular Density of the Optic Nerve Head in Active Thyroid-Associated Ophthalmopathy

**DOI:** 10.3389/fendo.2022.895186

**Published:** 2022-07-22

**Authors:** Pingyi Zhu, Zihui Liu, Yi Lu, Yu Wang, Danbin Zhang, Pinghui Zhao, Lulu Lin, Nimo Mohamed Hussein, Xiaozheng Liu, Zhihan Yan, Guanghui Bai, Yunhai Tu

**Affiliations:** ^1^ Wenzhou Medical University, Wenzhou, China; ^2^ Department of Radiology, The Second Affiliated Hospital and Yuying Children’s Hospital of Wenzhou Medical University, Wenzhou, China; ^3^ Department of Orbital and Oculoplastic Surgery, The Eye Hospital of Wenzhou Medical University, Wenzhou, China; ^4^ Department of Radiology, The First Affiliated Hospital, Zhejiang University School of Medicine, Hangzhou, China; ^5^ China-USA Neuroimaging Research Institute, The Second Affiliated Hospital and Yuying Children’s Hospital of Wenzhou Medical University, Wenzhou, China; ^6^ Wenzhou Key Laboratory of Basic Science and Translational Research of Radiation Oncology, Wenzhou, China

**Keywords:** active thyroid-associated ophthalmopathy, fractional low-frequency fluctuation amplitude, fMRI, resting state, optical coherence tomography angiography

## Abstract

**Purpose:**

To investigate changes in local spontaneous brain activity in patients with active thyroid-associated ophthalmopathy (TAO) and explore the relationship between such alterations and microvascular indices.

**Methods:**

Thirty-six active TAO patients with active phase and 39 healthy controls (HCs) were enrolled in this study. All participants underwent resting-state functional magnetic resonance imaging (rs-fMRI), neuropsychological tests, and ophthalmological examinations. The rs-fMRI-based fractional low-frequency fluctuation amplitude (fALFF) analysis methods were used to assess spontaneous brain activity in both groups. The structure (peripapillary retinal nerve fiber layer, pRNFL) and microvascular indices (the optic nerve head (ONH) whole image vessel density, ONH-wiVD, and peripapillary vessel density) were analyzed through optical coherence tomographic angiography imaging. The relationship between abnormal spontaneous brain activity and ophthalmological indices was analyzed using the Spearman’s rank correlation analysis.

**Results:**

Compared with HCs, active TAO patients had increased fALFF in the right inferior temporal gyrus (R.ITG) and left posterior cingulate gyrus (L.PCC), but decreased fALFF in the right calcarine (R.CAL). The fALFF values in L.PCC were positively correlated with peripapillary vessel density, whereas fALFF values in R.CAL were negatively related to peripapillary vessel density.

**Conclusions:**

This study demonstrates that changes in spontaneous brain activity of active TAO are accompanied by peripapillary microvascular variations. These results provide insights into the pathophysiological mechanisms of active TAO. In addition, the combination of fALFF values and peripapillary vessel density may be served as important references for better clinical decision making.

## Introduction

Thyroid-associated ophthalmopathy (TAO), commonly known as Graves’ ophthalmopathy or thyroid eye disease, is an organ-specific, progressive autoimmune inflammatory disease affecting the orbital. TAO is the commonest autoimmune orbital illness in adults (1, 2) and often involves both orbits, with an asymmetrical appearance (3, 4). The most common ophthalmic consequences in TAO patients are periorbital edema, upper eyelid retraction, diplopia, exophthalmos, dysfunctional eye movement, and impaired visual function (5–9).

TAO may cause permanent visual loss as well as significant facial disfigurement, significantly reducing the patient’s quality of life through restricted daily activity, social dysfunction, and diminished confidence (10, 11). Past studies have shown that TAO impairs the visual pathway and can harm cognitive processes (2–4, 10, 12, 13). Thus, there is an urgent need for a better understanding of the neuropathological mechanisms underlying TAO. Moreover, it is crucial to detect TAO early and prevent associated neuropsychic dysfunctions through prompt treatment, including anti-inflammatory therapy or decompression surgery.

The amplitude of low-frequency fluctuations (ALFF) produced by resting-state functional magnetic resonance imaging (rs-fMRI) is considered to be a direct indicator of the level of spontaneous brain activity (14, 15). ALFF has been extensively used in individuals with a range of ocular or metabolic illnesses, including glaucoma (16), neuromyelitis optica-spectrum disorders (17) and type 2 diabetes (18). However, ALFF is prone to artifacts generated by physiological noise (19). Fractional ALFF (fALFF) is another extensively used technique for determining the contribution of low-frequency oscillations to the overall frequency range (19) and it may be more sensitive than ALFF in detecting regional brain abnormalities since it is less prone to physiological noise artifacts (19). Moreover, fALFF based on rs-fMRI is considered one of the most reliable and reproducible fMRI parameters for assessing the brain’s physiological state (14, 15). However, few studies have investigated the value of fALFF in active TAO patients.

Optical coherence tomographic angiography (OCTA) is a novel angiographic technique based on optical coherence tomography (OCT) and can generate 3-dimensional microcirculation vascular maps and better displays of superficial and deep capillary layers of the retina without intravenous injection of a contrast agent. OCTA also generates quantitative data on retinal and choroidal vessels (20). Because it is safer, faster, and non-invasive, OCTA is widely used to diagnose and monitor retinal microvascular pathology in various diseases, including diabetic retinopathy, macular edema, and TAO (21, 22). Ophthalmological parameters, including microvascular detail and parapapillary retinal nerve fiber layer (pRNFL) thickness, can reflect the activity and severity of TAO (1, 23). Therefore, it is worth exploring whether ophthalmological parameters using OCTA correlate with altered fALFF values.

Here, we evaluated changes in local spontaneous brain activity of active TAO cases using fALFF, which can provide a more in-depth assessment of brain dysfunction. We also investigated whether fALFF values from aberrant brain regions correlate to the thickness of pRNFL and the vessels density of ONH, and offer insight into neuropathological mechanisms underlying active TAO.

## Methods

### Patients

This study involved patients with active TAO who were admitted to the Eye Hospital of Wenzhou Medical University, Zhejiang Province, China, from September 2019 to July 2021. All participants met the criteria of the European Thyroid Association/European Group (24). Active TAO was indicated by a 7-point clinical activity score (*CAS*) of ≥3 as described previous (25). The healthy controls (HCs) group was made of 39 healthy individuals recruited from community or the health care facility and matched with the TAO group for age, sex, and years of education by using the propensity score-matching (PSM) analysis (1:1 ratio, with nearest-neighbor matching or caliper width of 0.1 of the standard deviation of the logit). Based on Edinburgh Handedness Inventory, all participants were right-handed (26). Exclusion criteria were: (1) symptoms of other ocular diseases, e.g., strabismus, amblyopia, cataracts, and glaucoma; (2) history of eye surgery; (3) history of psychiatric or neurologic illness, e.g., depression and bipolar disorder; (4) alcohol or drug addiction; (5) brain structural abnormalities, e.g., tumors, trauma, and infection; (6) ineligibility for MRI scanning, e.g., due to implanted metal devices or cardiac pacemaker; (7) low image quality, e.g., motion artifacts, image distortion, which would affect the accuracy of fMRI analysis.

All participants underwent ophthalmological examination, neuropsychological tests, and MRI scanning within a week. Neuropsychological tests included the Beck Depression Inventory-II (BDI-II) (27) and Insomnia Severity Index (ISI) (28). Records on the participants’ general medical history and ophthalmology history were retrieved. Participants in the patients group were asked about the courses of hyperthyroidism and TAO. A total of 36 patients with active TAO (17 females and 19 males) and 39 HCs (21 females and 18 males) were finally enrolled into the study. The enrollment flowchart has been presented in the **Supplementary Figure 1**.

Ethical approval for the study was granted by the research ethics committee of the Eye Hospital of Wenzhou Medical University(2020-142-K-127). This study complied with the Declaration of Helsinki guidelines. All study participants gave written informed consent.

### MRI Acquisition

MRI examination was done using a 3.0 Tesla scanner (GE Healthcare’s Discovery 750, USA) equipped with a 32-channel phase array head coil. The MRI room was completely dark during the tests. Participants were instructed to maintain a steady position, and kept eyes closed while awake. To protect the auditory system, participants were asked to wear noise-canceling earplugs. A sponge pad was used to minimize head movement. Before rs-fMRI scanning, routine head MRI scans, including T1, T2, T2Flair, and diffusion-weighted images, were obtained and analyzed by an expert radiologist to rule out structural brain abnormalities. High-resolution volumetric 3-D T1-weighted imaging data were acquired on a 3D BRAVO sequence in the sagittal orientation using the following parameters: slices = 188, field of view (FOV) = 256mm×256mm, matrix size = 256×256, slice thickness = 1mm, repetition time (TR) = 7.7ms, echo time (TE) = 3.4ms, flip angle (FA) = 9°, no gap. rs-fMRI images were acquired for the whole brain in an axial orientation with a gradient echo planar imaging sequence using the following parameters: slices = 54, FOV = 216mm×216mm, TE = 30ms, TR = 2500ms, flip angle (FA) = 90°, matrix size = 72×72, slice thickness = 3mm, no gap.

### MRI Data Preprocessing

rs-fMRI data preprocessing was done using Data Processing Assistant for Resting-State fMRI Advance (DPARSFA, http://www.restfmri.net), a software package based on statistical parametric mapping (SPM8, http://www.fil.ion.ucl.ac.uk/spm). The first 10 volumes were eliminated in order to reduce the environmental noise and to achieve a constant level of magnetization. Remaining volumes were then corrected for slice timing and head motion (head translation or rotation by 3mm in 3 directions or axes). To minimize confusing signals, regression analysis was used (the white matter, cerebral-spinal fluid, and Friston 24 head motion parameters). Images were then normalized in the standard Montreal Neurological Institute (MNI) space using a resampling resolution of 3×3×3mm^3^ and spatially smoothed with a 6mm full width at half maximum (FWHM) kernel.

### fALFF Analysis

The fALFF were examined using the DPARSFA software based on the preprocessed data. To acquire a power spectrum, preprocessed time series were transformed into the frequency domain using the fast Fourier transform (FFT) (29). The square root of each frequency of the power spectrum was calculated and the average square root of the spectrum in the range of 0.01-0.08 Hz for each voxel analyzed as ALFF (29). To eliminate interference from respiratory and cardiac signals, ALFF values were divided by the sum of the amplitudes across the entire frequency band to get the fALFF (19).

### Ophthalmological Examinations

**Figure 1:**
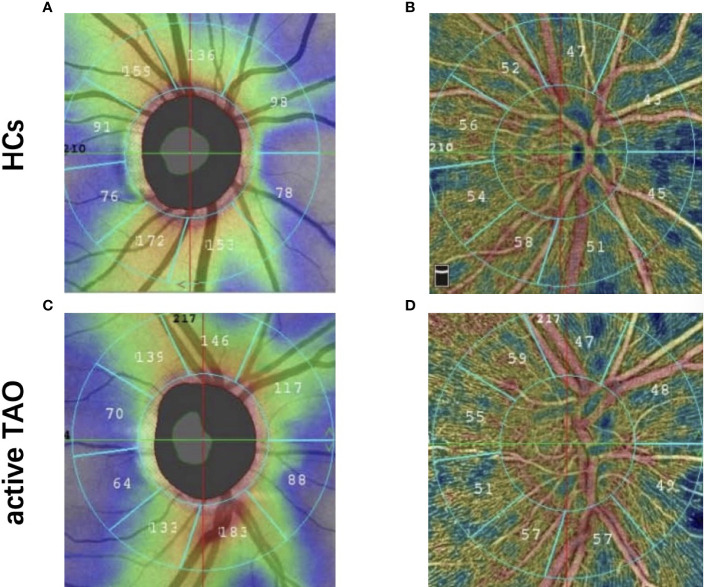
Representative OCT-A image between two groups. Peripapillary retinal nerve fiber layer thickness image in healthy eye **(A)** and active TAO **(B)** eyes. Optic nerve head whole image vessel density and peripapillary vessel density map in healthy eye **(C)** and active TAO **(D)** eyes.

Each individual underwent complete ophthalmological examinations, which included axial length (AL), intraocular pressure, slit lamp examination, best-corrected visual acuity (BCVA), and standard automated perimetry using the 30-2 program of the Humphrey Visual Field Analyser (Carl Zeiss Meditec, Dublin, CA, USA), peripapillary retinal nerve fibre layer (pRNFL) thickness using the SD-Optical Coherent Tomography (RTVue XR Avanti, Optovue Inc. Fremont, CA. USA), and vascular density calculated using the manufacturer’s angio-metric software (**Figure 1**). The ONH scanning region was classified into 2 parts using an ONH grid, including whole image and peripapillary fields. The default quantified vascular layer is superficial retinal layers which extend from the inner layer membrane to the retinal nerve fiber layer posterior boundary. Vascular density was defined as area of pixels occupied by retinal vessels divided by total area of the whole image. To rule out eye disorders, all patients and HCs were checked by an ophthalmologist with 8 years of experience in active TAO treatment. Although both eyes were eligible for investigation, final analysis involved only data from the right eye.

### Statistical Analysis

Two-sample t-test was done using the REST software to compare fALFF differences between active TAO and the HCs, with age and sex as nuisance covariates. AlphaSim multiple comparison correction was done using 3dClustSim on AFNI (Analysis of Functional NeuroImages software) software (http://afni.nimh.nih.gov). The significance threshold was set at uncorrected p<0.005, with a voxel number >25, corresponding to corrected p<0.05.

The Kolmogorov-Smirnov test was used to normalize continuous variables. The Mann-Whitney U test was used to compare non-normally distributed variables. Normally distributed data were compared using an independent-samples t-test. Differences between two groups of categorical variables were compared using Chi-squared testing. Association between fALFF values and ophthalmological data from active TAO cases was assessed using Spearman’s rank correlation analysis. Statistical analyses were done on SPSS version 22 (IBM) and Prism version 7 (GraphPad). P<0.05 was considered statistically significant.

## Results

### Demographic and Ophthalmological Characteristics Between Active TAO and HCs

The participants’ demographic and ophthalmological features are shown on **Table 1**. The active TAO and HC groups did not differ significantly with regards to age, education level, gender, and pRNFL thickness (p>0.05). No notable differences were observed in the BDI-II and ISI (p>0.05) between active TAO and HCs group. Relative to the HCs group, the active TAO group had a significantly lower BCVA (p<0.001) and onh-wiVD (p<0.05). Additionally, relative to HCs, active TAO patients exhibited significantly higher vessel density in the peripapillary (p<0.001) and IOP (p<0.001).

**Table 1:** Demographic and ophthalmological characteristics between active TAO and HCs group.

Characteristics	active TAO (n=36)	HCs (n=39)	*P-*value	
	N (%) or x^-^ ± s or M (P_25_, P _75_)			
**Demographic**				
Age (years)	46.6 ± 11.6	47.1 ± 11.3		0.875^c^
Gender				0.566^b^
-male	19 (52.8)	18 (46.2)		
-female	17 (47.2)	21 (53.8)		
With a history of				
-smoking	5 (13.9)	4 (10.3)		0.629^b^
-alcohol drinking	5 (13.9)	5 (12.8)		0.892^b^
-hypertension	5 (13.9)	6 (15.4)		0.855^b^
-diabetes mellitus	1 (0.1)	2 (0.1)		0.604^b^
**Ophthalmological**				
BCVA	1.0 (0.85-1.0)	1.2 (1.0-1.2)		**<0.001**∗^b^
AL, mm	23.0 (21.0-24.5)	23. 2(22.7-24.5)		0.213^a^
IOP, mmHg	17.6 (12.7-21.5)	12.6 (10.9-14.2)		**<0.001**∗^a^
pRNFL thickness (um)	114.4 ± 14.5	114.9 ± 11.9		0.869^c^
vessel density (%)				
-ONH-wiVD	56.7 (54.3-57.6)	58.6 (56.1-59.3)		**0.042**∗^a^
-Peripapillary	58.2 ± 3.1	52.8 ± 3.0		**<0.001**∗^c^

a p value with Mann–Whitney test.

b p value with Chi-square test.

c p value with independent-samples t-test.

*Significant at p < 0.05.

TAO, Thyroid-Associated Ophthalmopathy; HCs, healthy controls; BCVA, Best-corrected visual acuity; AL, axial length; IOP, intraocular pressure; pRNFL, peripapillary retinal nerve fiber layer; ONH-wiVD, optic nerve head whole image vessel density.

### Differences of fALFF Values Between Active TAO and HCs

**Figure 2:**
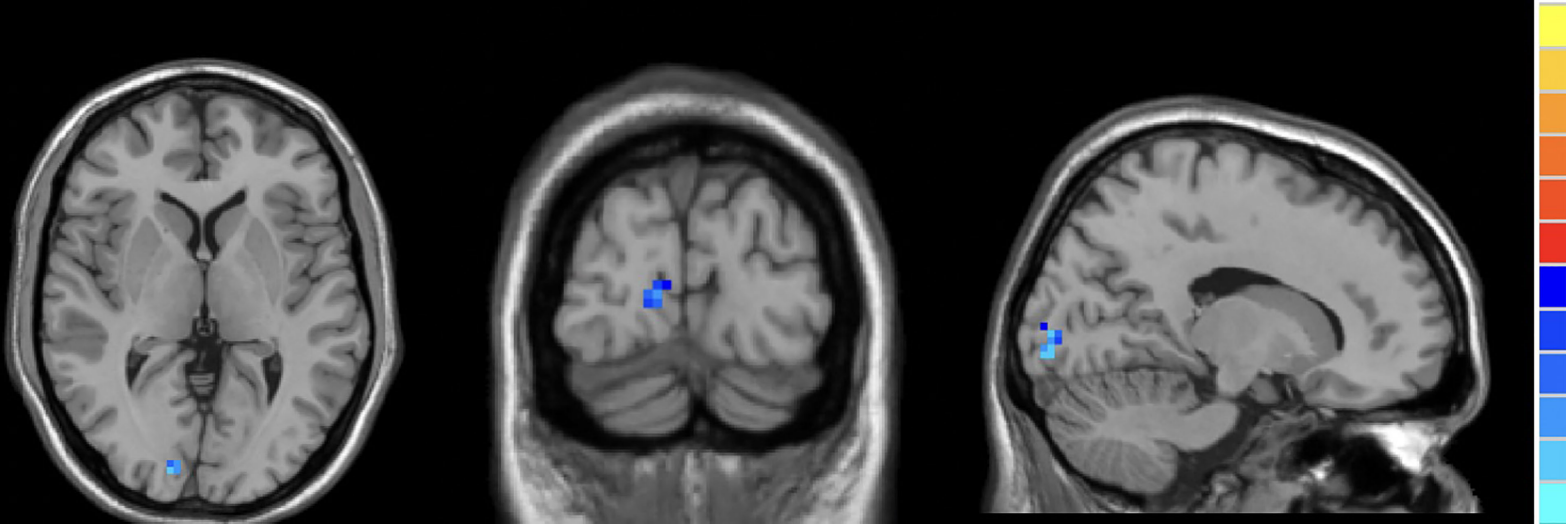
In comparison with HCs, active TAO patients showed decreased fALFF in the right calcarine.

**Figure 3:**
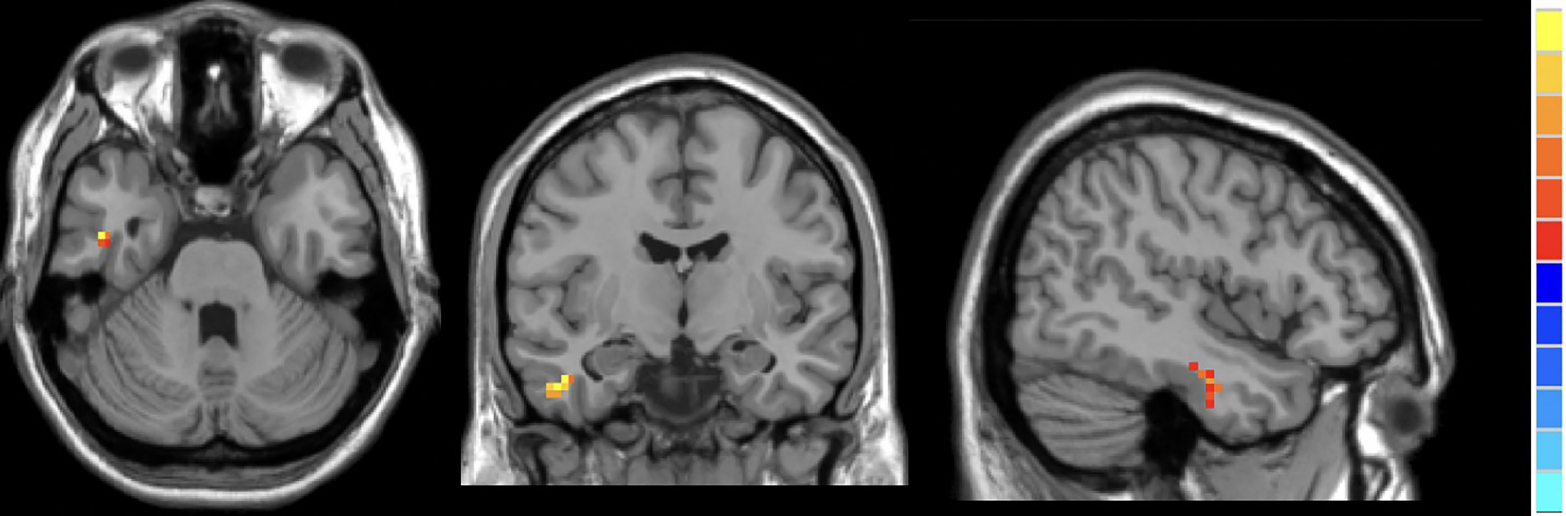
In comparison with HCs, active TAO patients showed increased fALFF in the right inferior temporal gyrus.

**Figure 4:**
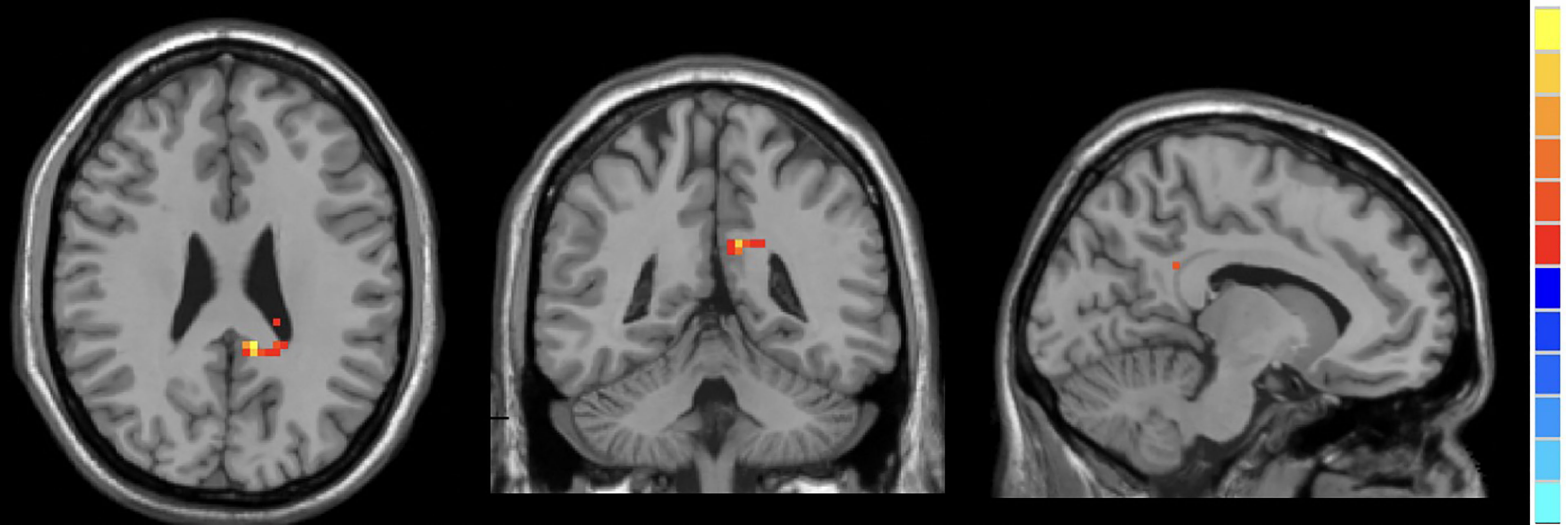
In comparison with HCs, active TAO patients showed increased fALFF in the left posterior cingulate gyrus.

Compared to HCs, active TAO patients exhibited increased fALFF in the right inferior temporal gyrus (R.ITG) and left posterior cingulate gyrus (L.PCC), and reduced fALFF in the right calcarine (R.CAL, **Table 2**; **Figures 2**–**4**).

**Table 2:** Brain regions with significant differences of fALFF in subjects with active TAO compared to HCs.

Brain regions	Cluster size	T values	MNI coordinates		
			X	y	z
TAO>HCs					
R.ITG	33	3.4880	51	-9	-33
L.PCC	27	4.1549	-9	-42	27
TAO<HCs					
R.CAL	29	-3.4305	12	-96	-6

fALFF, fractional Amplitude of Low Frequency Fluctuation; TAO, thyroid-associated ophthalmopathy; HCs, healthy controls; MNI, Montreal Neurological Institute; R.ITG, right inferior temporal gyrus; L.PCC, left posterior cingulate gyrus; R.CAL, right calcarine.

### Correlations Between fALFF Values and Ocular Variations

**Figure 5:**
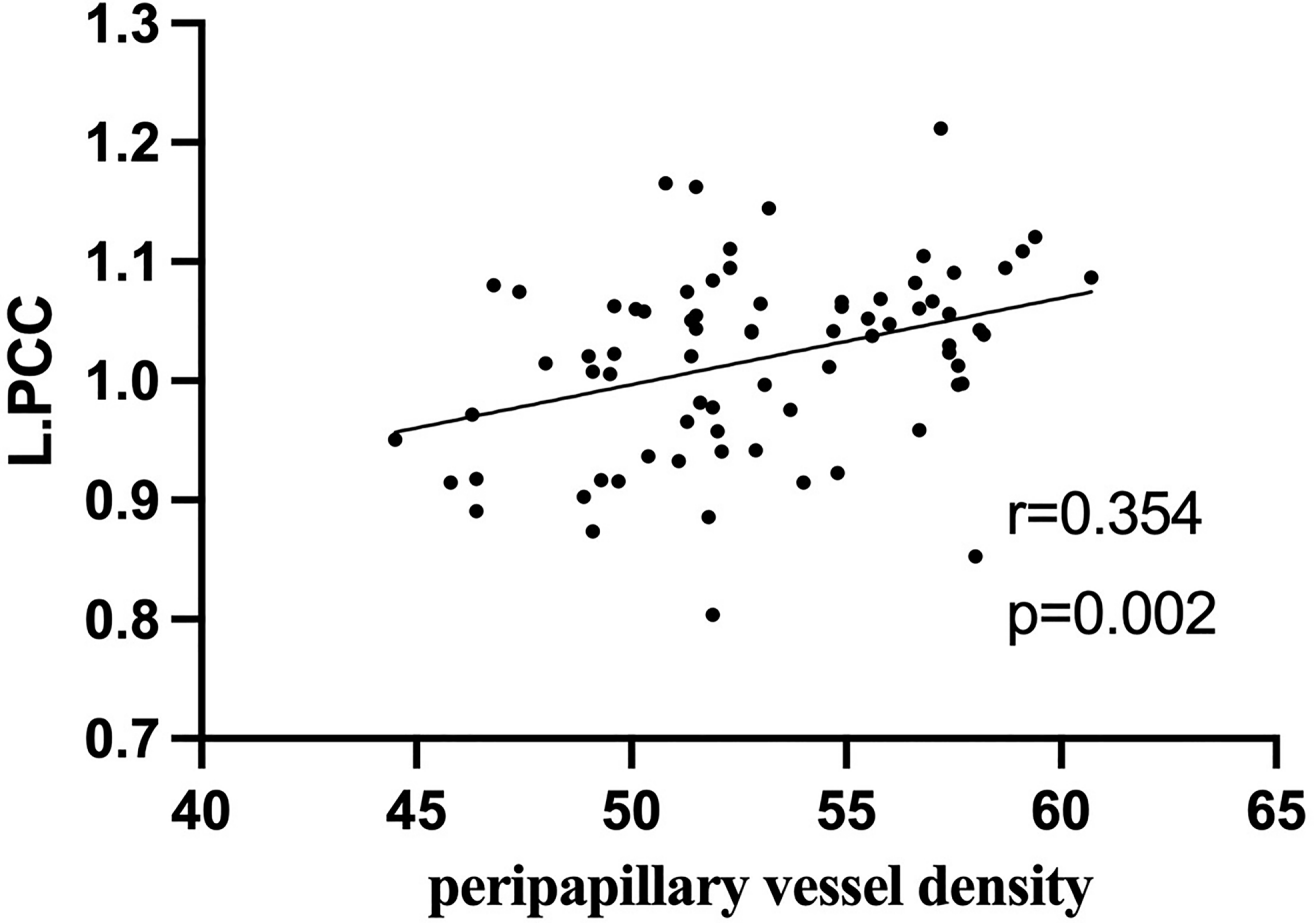
In active TAO group, the fALFF values in L.PCC were positively correlated with peripapillary vessel density (r = 0.354, p = 0.002).

**Figure 6:**
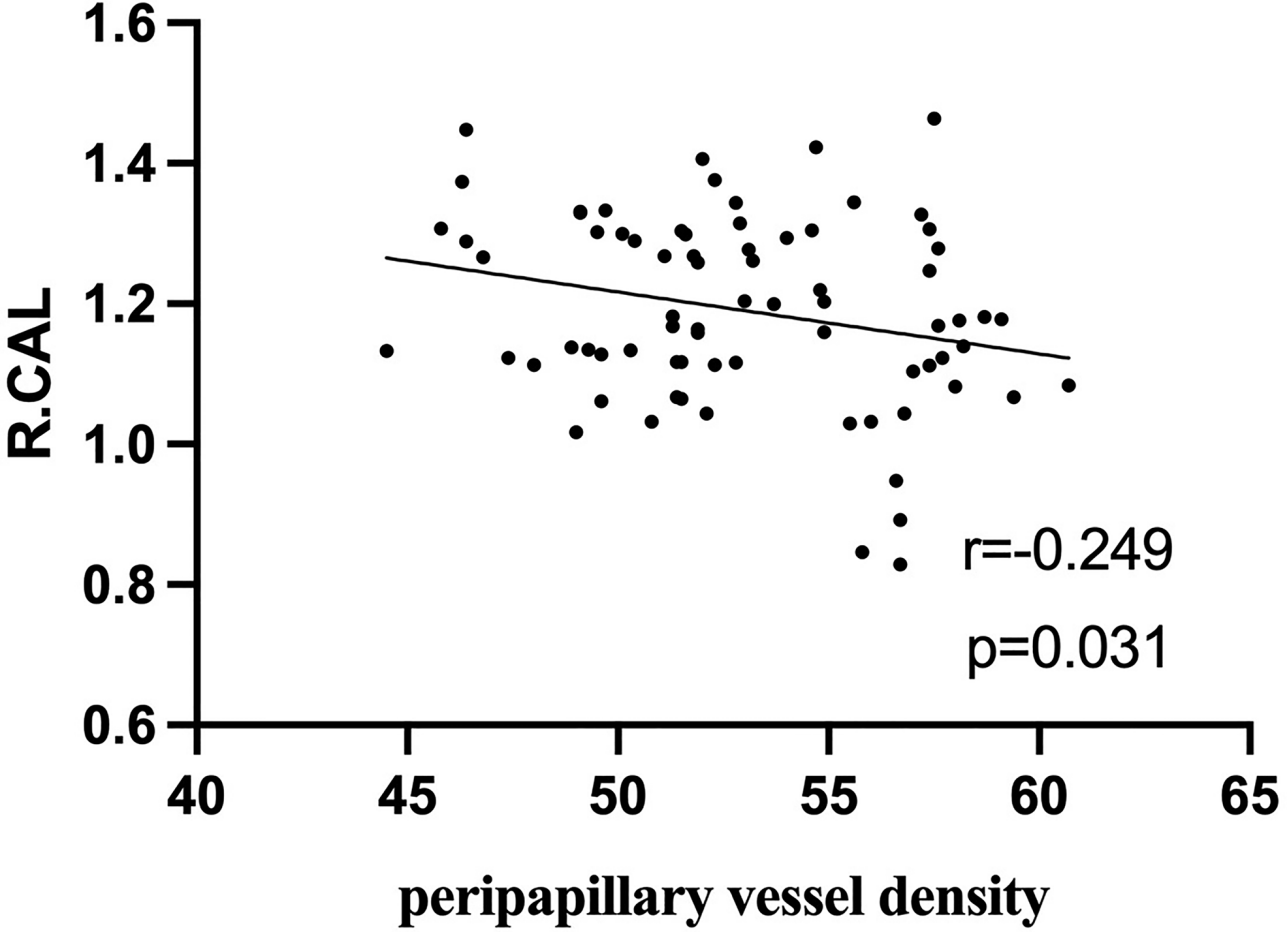
In active TAO group, the fALFF values in R.CAL were negatively correlated with peripapillary vessel density (r = -0.249, p = 0.031).

In active TAO group, fALFF values in L.PCC positively correlated with peripapillary vessel density (r=0.354, p=0.002, **Figure 5**). fALFF values in R.CAL negatively correlated with peripapillary vessel density (r=-0.249, p=0.031, **Figure 6**). However, there was no significant correlation between altered fALFF values and ONH-wiVD or pRNFL thickness (p>0.05). In HC group, there was no significant correlation between abnormal fALFF values and ocular variation (p>0.05).

## Discussion

Although TAO impairment is well documented at the optic nerve and retinal microvascular levels, it is poorly understood at the whole brain activity level. Our data indicate that active TAO patients exhibit significant alternation in spontaneous neural activity in brain regions mainly associated with visual and visual-related functions. Furthermore, fALFF values in abnormal brain regions significantly correlated with peripapillary microvascular impairment. To the best of our knowledge, this is the first study that provided novel insights into regional abnormalities in fALFF utilizing rs-fMRI processing technique, detecting the correlations between altered spontaneous neuronal activity across the entire brain and peripapillary microvascular variations in patients with active TAO, and developing a neuroimaging biomarker for assessing the disease severity and activity.

In this study, we demonstrated that patients with active TAO presented increased peripapillary vessel density compared with the healthy controls. According to existing literatures, acute inflammation may be the main reason, which increases orbital arterial blood flow velocities (30, 31). Consistently, Lei Ye et all (22) also observed that TAO patients had increased retinal microvascular density. However, Jian H et all (32) didn’t find a significant difference in peripapillary vessel density between the active non-dysthyroid optic neuropathy (DON) TAO patients and the normal control group, while TAO patients with DON had a significant decreased peripapillary vessel density in comparison to other patients. Researchers postulated that this phenomenon was the consequence of increased venous pressure and choroidal vessel resistance due to elevated intra-orbital pressure. From our prospective, the inconsistent results across studies may be attributed to the differences in severity and activity of the disease.

Relative to HCs, active TAO patients exhibited lower spontaneous neuronal activity in the R.CAL located in the occipital lobe, which is consistent with previous findings (2, 10, 12). The occipital lobe is primarily involved in visual formation and functional visual perception processes (33), and it is where visual information is integrated with information from sensory systems, including the auditory system (34). CAL is regarded to be the primary visual cortex since it receives direct visual input from the eyes *via* thalamic relays (34). Additionally, CAL is included in the Brodmann’s region 17(BA17). Previous findings show that cortical visual stimulus generators induce steady-state responses specific to occipital areas *via* visual stimulus streams (35). More specialized, centers of gravity were located in BA17 (35), which revealed that BA17 is crucial for basic visual processing. BA17 receives visual signals from the lateral geniculate nucleus and transmits them to the secondary visual cortex (36). A similar finding revealed that BA17 is important in fundamental visual processing, including visual orientation, spatial frequency, and color perception (16). Our findings of decreased spontaneous neural activity in the R.CAL imply impairment of visual information processing in patients with active TAO, which is consistent with previous findings (16).

Our analyses also revealed increased spontaneous brain activity in R.ITG, which is located in the temporal lobe. Temporal lobe regions are critical for visual information processing and receive substantial inputs from the visual cortex for visual object recognition and spatial localization (34). Moreover, the ITG is considered to be the final location of the ventral cortical visual system, which serves as a link between auditory and visual processing, perception, and memory (37). Notably, ITG is involved in high cognitive functions including emotional regulation (38) and social cognition (39, 40). Previous studies on TAO patients indicate a clear tendency toward anxiety and depression (2, 10). Thus, increased spontaneous neural activity in R.ITG may underlie the dysfunctional visual and emotional regulation in patients with active TAO, contributing to emotional lability, cognitive dysfunction, and increased prevalence of suicidal tendencies.

Here, we find that compared to HCs, active TAO patients exhibited higher L.PCC fALFF values. PCC and ITG form a pivotal hub of the default mode network (DMN) (41, 42). The DMN is involved in the maintenance of baseline brain activities associated with spontaneous cognition, self-awareness, monitoring of the external environment and interactive modulation between internal mind activities and external tasks, which are critical for cognitive performance (42–44). Abnormal spontaneous activities in numerous DMN brain regions is observed in patients with endocrine diseases, such as painful diabetic neuropathy (18), who also demonstrate clear proclivity for anxiety and depression. Accordingly, we could deduce that abnormal spontaneous neuronal activities in the DMN brain regions of patients with active TAO may contribute to cognition dysfunction. Furthermore, active TAO patients are more inclined to irritability and anxiety (45), which may be associated with DMN dysfunction.

ONH vessel density and pRNFL thickness are measured by OCT-A and are useful in identifying TAO patients with DON, the most common vision-threatening condition in TAO and damaged optic nerves (23). It is reported that retinal nerve fiber layer thickness correlates with vascular density and the disease activity of TAO (1). Past studies show that altered fALFF in specific brain regions (left cuneus, bilateral superior frontal gyrus) correlates with RNFL thickness in patients with primary angle-closure glaucoma, suggesting that these finding are conducive to assessing the disease severity (16). A similar finding indicated that functional connectivity density correlates with pRNFL thickness in patients with neuromyelitis opticaspectrum disorders (17). However, to our knowledge, the correlation between fALFF and pRNFL thickness or ONH vascular density in active TAO patients has not been previously investigated. In this study, correlation analysis revealed that the fALFF value of L.PCC positively correlated with peripapillary vessel density. Moreover, we found negative correlation between fALFF values in R.CAL with peripapillary vessel density. These results indicate that combining fALFF values from these brain regions with vessel density in the peripapillary may indicate disease severity in active TAO patients, as well as their involvement in DON.

Several limitations of current research should be recognized. First, neuropsychological tests in our study only included BDI-II and ISI. Future research should include more detailed and comprehensive neuropsychological tests to further confirm association between spontaneous brain activity with cognitive changes. Secondly, our sample size was relatively small. Further studies with a larger sample size could strengthen the statistical power and verify these results. Thirdly, the study was a cross-sectional design. Therefore, a longitudinal* *follow*-*up* *of these subjects may provide more information and reinforce the present interpretation.

## Conclusion

In summary, our results show that active TAO patients exhibit significant alternations of spontaneous neural activity in brain regions associated with visual and visual-related functions. Additionally, fALFF values in abnormal brain regions significantly correlated with peripapillary microvascular impairment. These abnormalities may provide a clue to reveal neuropathological mechanisms of active TAO. The fALFF results from certain brain regions together with peripapillary vessel density may be served as important references for better clinical decision making.

## Data Availability Statement

The raw data supporting the conclusions of this article will be made available by the authors, without undue reservation.

## Ethics Statement

The studies involving human participants were reviewed and approved by the research ethics committee of the Eye Hospital of Wenzhou Medical University. The patients/participants provided their written informed consent to participate in this study.

## Author Contributions

All the authors included in this paper fulfil the criteria of authorship. GB and YT designed the study and had full access to all data in this study. PYZ, ZL, YL and YW enrolled the participants. DZ, PHZ and LL collated all the preoperative and postoperative data. XZL processed the data. PYZ drafted the manuscript. NH, ZY, GB and YT reviewed and edited the manuscript. All authors read and approved the manuscript.

## Funding

This research was supported by the National Natural Science Foundation of China (Grant Nos.81771914, 82071902, 82071993), Natural Science Foundation of Zhejiang Province (nos. LY19H180003) and Wenzhou Science and Technology Bureau in China (No. Y20180112).

## Conflict of Interest

The authors declare that the research was conducted in the absence of any commercial or financial relationships that could be construed as a potential conflict of interest.

## Publisher’s Note

All claims expressed in this article are solely those of the authors and do not necessarily represent those of their affiliated organizations, or those of the publisher, the editors and the reviewers. Any product that may be evaluated in this article, or claim that may be made by its manufacturer, is not guaranteed or endorsed by the publisher.

## Supplementary Material

Supplementary Figure 1 Flowchart of patient enrollment.
