# Supplementary figures and images for: Alterations in Spontaneous Neuronal Activity and Microvascular Density of the Optic Nerve Head in Active Thyroid-Associated Ophthalmopathy
Source: Front Endocrinol (Lausanne). 2022 Jul 22;13:895186. doi: 10.3389/fendo.2022.895186 (PMC9354054; doi:10.3389/fendo.2022.895186)

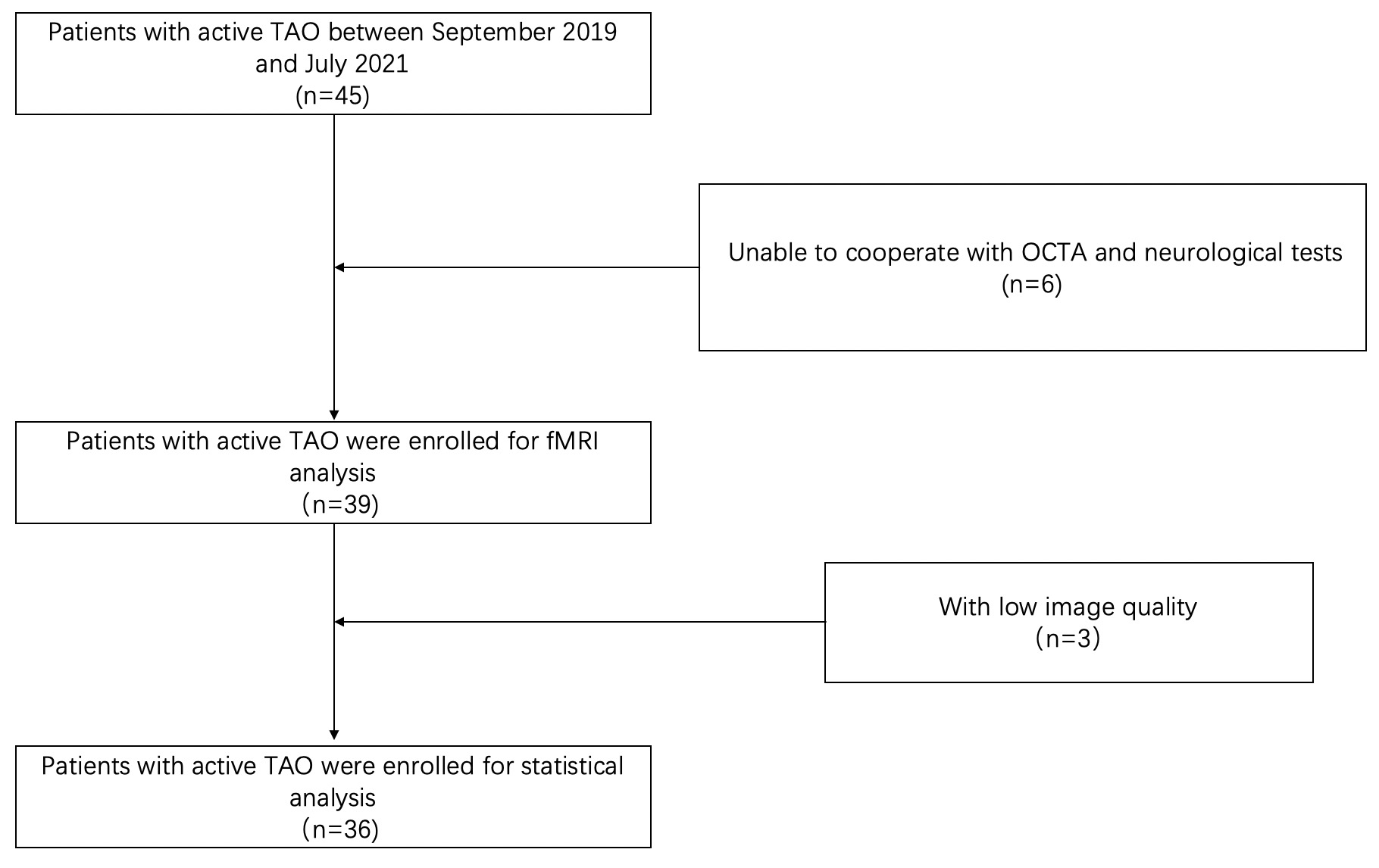

Supplement: Supplementary Figure 1 — Flowchart of patient enrollment. [file Image_1.jpeg]
